# Photovoltaic module dataset for automated fault detection and analysis in large photovoltaic systems using photovoltaic module fault detection

**DOI:** 10.1016/j.dib.2024.111184

**Published:** 2024-12-02

**Authors:** Rotimi-Williams Bello, Pius A. Owolawi, Etienne A. van Wyk, Chunling Du

**Affiliations:** Department of Computer Systems Engineering, Faculty of Information and Communication Technology, Tshwane University of Technology, South Africa

**Keywords:** Anomalies, Cracks, Hotspots, Shadings, Solar cells

## Abstract

Solar energy has become the fastest growing renewable and alternative source of energy. However, there is little or no open-source datasets to advance research knowledge in photovoltaic related systems. The work presented in this article is a step towards deriving Photo-Voltaic Module Dataset (PVMD) of thermal images and ensuring they are publicly available. The work provides a PVMD dataset comprising a total of 1000 self-acquired and augmented images. The dataset includes both permanent and temporal anomalies, namely Hotspots, Cracks, and Shadings. The dataset was collected on September 5, 2024 at the Soshanguve South Campus, Tshwane University of Technology, South Africa using DJI Mavic 3 Thermal's high-resolution thermal and visual imaging capabilities. DJI Mavic 3 Thermal coupled with its advanced flight features makes it an excellent tool for precise and efficient inspections of PV systems. The laboratory experiment performed on the dataset lasted one week. The work aims to provide supervised dataset good enough to support research method in providing a comprehensive and efficient approach to monitoring and maintaining large PV systems. Extensive analysis of the thermal data reveals the anomalies as indicative of faults in the solar cells of PV module, thereby opening up advancement in solar energy research. Because the data comes from a single-day collection and one week laboratory experiment, it makes the data more suitable for testing algorithms designed for fault detection. The dataset is publicly and freely available to the scientific community at 10.17632/5ssmfpgrpc.1

Specifications Table

**Table:** 

Subject	Computer Science, Computer Engineering
Specific subject area	Computer Vision, Image Processing, Image Classification, Machine Learning, Photovoltaic System, Solar Cell Faults
Type of data	Raw data, images
Data collection	The data were collected using DJI Mavic 3 Thermal's high-resolution thermal and visual imaging capabilities. The dataset includes both permanent and temporal anomalies, namely hotspots, cracks, and shadings. The RGB images which were originally of different dimensions due to drone employed, unfriendly and unstable environmental conditions were trimmed to size 512×512×3 (3 keeps the color information) and augmented
Data source location	The Soshanguve South Campus, Tshwane University of Technology, South Africa
Data accessibility	Repository name: Mendeley data Data identification number: 10.17632/5ssmfpgrpc.1 Direct URL to data: https://data.mendeley.com/datasets/5ssmfpgrpc/1 Instruction for accessing these data: No restriction to data accessibility
Related research article	None

## 1 Value of the Data

• The dataset assists in identifying the best locations for solar power generation and assessing the performance of PV fault forecasting systems.
• The dataset assists in examining a wide range of issues such as solar adoption trends and the performance and reliability of solar energy generation facilities.
• The dataset enables predictive maintenance, that is, it assists in predicting when solar energy equipment will fail, allowing solar energy providers to proactively perform maintenance and avoid unexpected downtime.
• The dataset assists in data analytics for financial planning in solar energy projects, and by analyzing historical data and market trends, future solar energy production and maintenance costs can be predicted.
• The dataset reveals the thermal anomalies as indicative of faults in the solar cells of PV module and thus opening up advancement in solar energy research.
• The dataset is publicly available for testing algorithms designed for fault detection in solar cells of PV module.

## 2 Background

As demand for electricity increases, there is a need to also consider its stability. The experiment performed in [1] on PV systems reveals faults as potential factor that can contribute to power instability or total loss. Traditional method of repairing or solving any identified faults in PV systems (especially large PV systems) are time-consuming, inaccurate, skills-dependent, etc. [2], thereby necessitating automated monitoring for fault detection and analysis in large PV systems. Considering the above-mentioned issues, the PVMD dataset [3] was collected, and this was by taking faulty PV module images from the Soshanguve South Campus, Tshwane University of Technology, South Africa using DJI Mavic 3 Thermal's high-resolution thermal and visual imaging capabilities. The thermal imaging was performed by using DJI Mavic 3 Thermal drone equipped with an integrated thermal camera capable for advanced aerial thermal imaging and inspection tasks. We simulated external defects on the system by placing materials such as polystyrene behind the PV panel, adhesive paper on the front glass, and a small piece of gum. Python, along with libraries such as Flask, OpenCV, NumPy, and Matplotlib, was used to analyze and process the images for PV system performance and fault detection. The libraries were installed using pip commands, facilitating the implementation of the image processing tasks such as thermal anomaly detection.

## 3 Data Description

The work provides a PVMD dataset comprising a total of 1000 self-acquired and augmented images. The dataset includes both permanent and temporal anomalies such as hotspots, cracks, and shadings. The RGB images which were originally of different dimensions due to unfriendly and unstable environmental conditions were trimmed to size 512×512×3 (3 keeps the color information) and augmented. A file named PVMD dataset, comprising the dataset was stored in Mendeley data, a free and secure cloud-based communal repository where data can be stored, ensuring easy sharing, accessibility and citing. The Mendeley data comprises three folders, namely Hotspots, Cracks and Shadings. The folder name is the name of the particular anomaly in it. The Hotspots folder comprises 350 thermal generated images; the Cracks folder comprises 350 thermal generated images; and the Shadings folder comprises 300 thermal generated images. As earlier mentioned, the work aims to provide supervised dataset good enough to support scientific research in providing a comprehensive and efficient approach to monitoring and maintaining large PV systems. A concise description of the three anomalies is presented as follows.

### 3.1 Hotspots anomaly

**Fig. 1:**
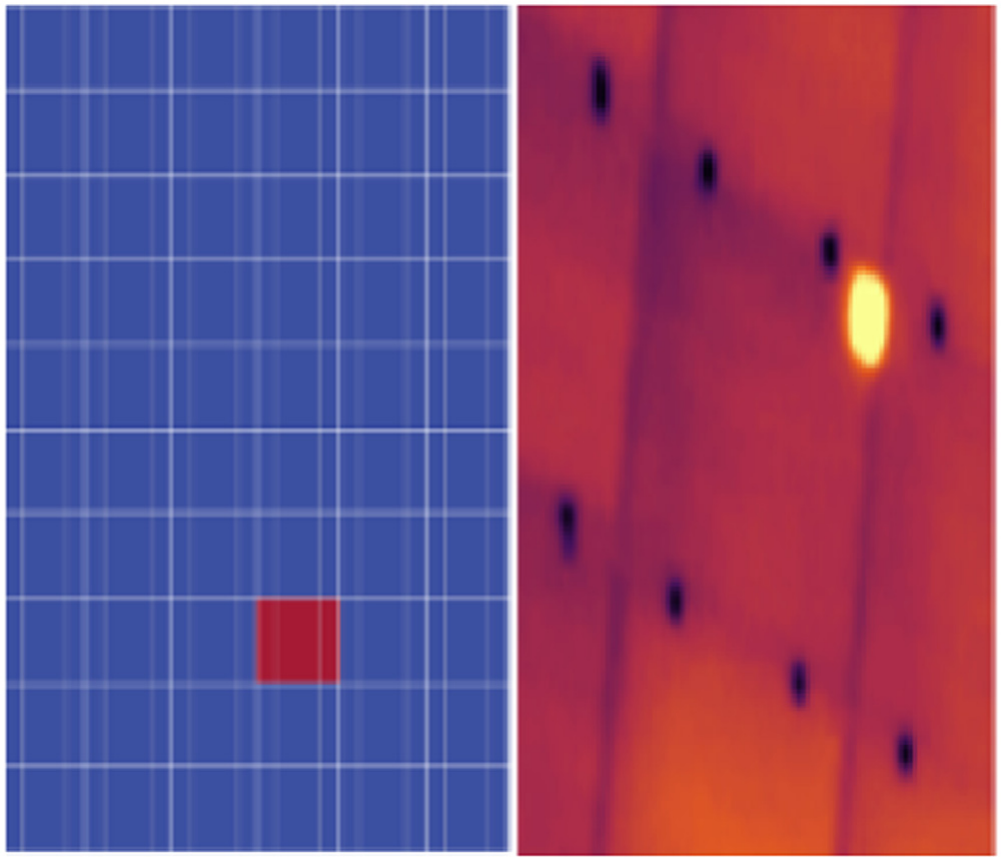
Sample PV module affected by the Hotspots thermal anomaly [3].

Usually, Hotspots are single heated conspicuous cells. Hotspots are indicators of various underlying issues with PV module, such as shading and soiling. Multiple hotspots indicate multiple hot cells in the module. The best method to spot shading and soiling as likely root causes of the issues is RGB visual imagery. The common method of resolving the conspicuous spotted shading and soiling on the surface of the module is by cleaning and trimming any overgrown plants. Energy generated by other cells in the modular string is easily driven away by a shaded cell, which acts as a resistor. Under certain circumstances, the design of the components of the PV module makes the shading permanent [1]. Further troubleshooting may be recommended for identification of hidden causes if the fault goes beyond soiling or shading. Few of the hidden causes are delamination, cell shunting, cell mismatch, etc. Ground-based visual inspection of thermal imaging can be an overall solution to some of the above-mentioned issues [2]. Fig. 1 shows a sample PV module image which has been affected by the Hotspots thermal anomaly. A total of 350 images were collected and processed for this category.

### 3.2 Cracks anomaly

**Fig. 2:**
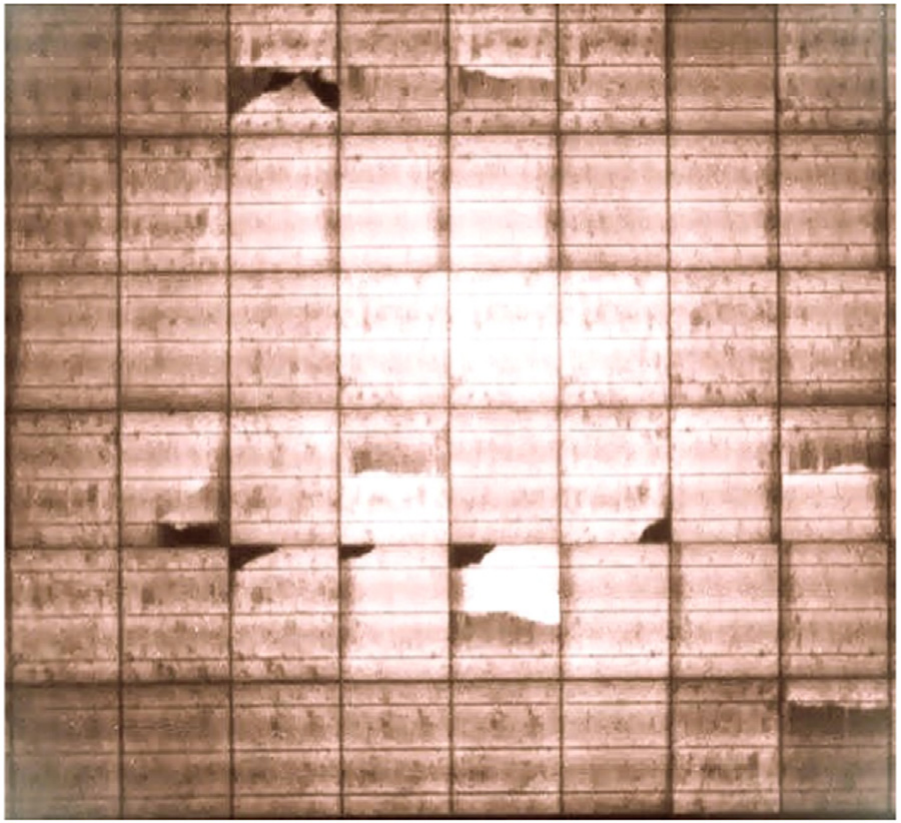
Sample PV module affected by the Cracks thermal anomaly [3].

Cell cracking refers to occurrence of microcracks among the solar cells in PV module. Several factors can cause the occurrence of cell cracking, namely manufacturing stress, environmental factors, transportation and handling. There are severe probable consequences of cell cracking for solar PV systems, including reduced efficiency, lower energy output, shortened module lifespan, etc. Cell cracking has many challenges; among which is its difficulty to detect and analyze. If this issue remains undetected, it can lead to occurrence of performance degradation [1]. To visualize the microcracks, specialized technological tools, such as electroluminescence (EL) imaging, is required. The major techniques for effective identification of cell cracking in solar cells are EL imaging and infrared (IR) thermography. The impact of cell cracking on the ROI for solar projects includes reduced efficiency, lower energy output, and shortened module lifespan. This can affect the optimal performance of solar PV systems, thereby reducing overall satisfaction [2]. Cell cracking of solar PV systems can be addressed by ensuring the following: (a) Manufacturing process of solar PV systems is enhanced, (b) Solar PV systems are handled with care during transportation and installation, (c) Carrying out regular inspections on the solar PV systems, (d) Remote monitoring of the solar PV systems, (e) Periodic research and development. Fig. 2 shows sample PV module affected by the Cracks thermal anomaly. A total of 350 images were collected and processed for this category.

### 3.3 Shadings anomaly

**Fig. 3:**
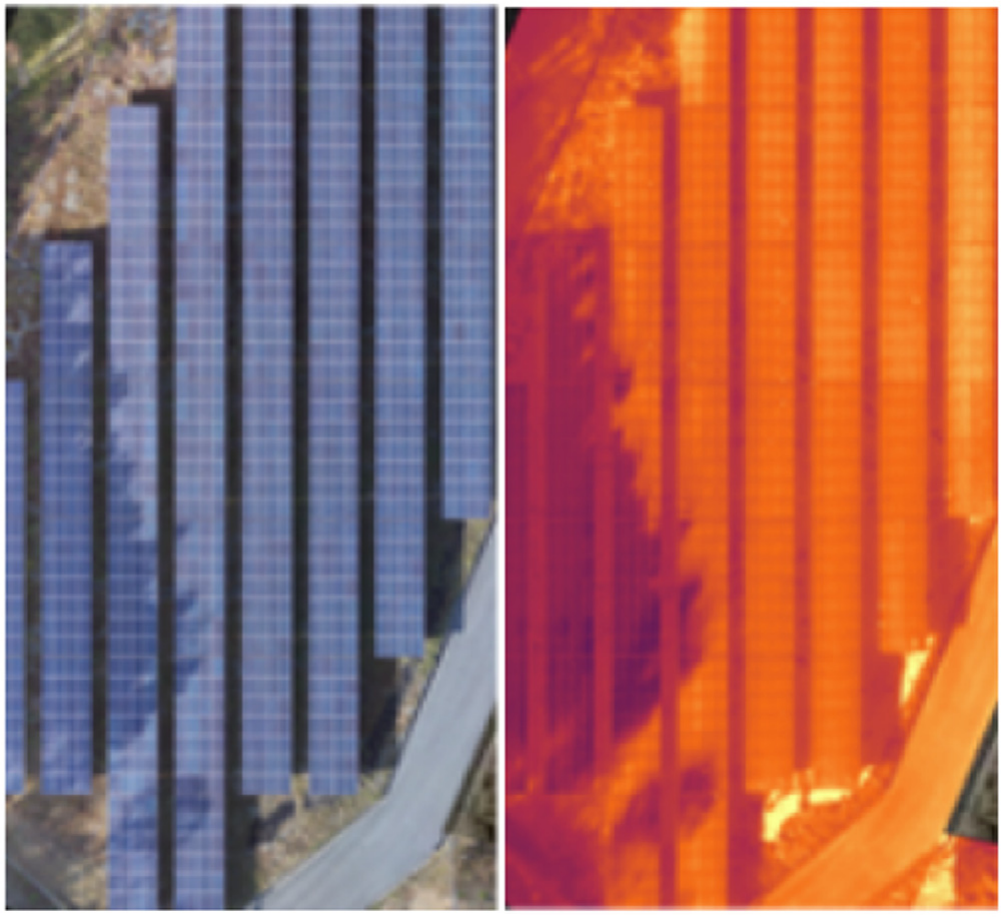
Sample PV module affected by the Shadings thermal anomaly [3].

Shadings are not hotspots; rather, they are cold spots, and this is due to objects’ shadings such as trees, poles (could be electric pole), fences, etc. Under this scenario, there is very high expectation of these shadings turning into hotspots sooner or later if the shadings persist. However, shadings that are temporary shadings either disappear or change position as sun changes position. As aforementioned, Shadings can be caused by any objects such as trees, poles (could be electric pole), fences, etc. The best remedy for mitigating shadings is by removing the materials causing shadowing [1]. Accurate estimation of power loss due to Shadings cannot be easily obtained since Shading anomalies are temporary anomalies and disappear or change position as sun changes position. Nevertheless, there is a need for reschedule analysis of the panels if the shadings and the overheating of the panels persist. Fig. 3 shows sample PV module affected by the Shadings thermal anomaly. A total of 300 images were collected and processed for this category.

## 4 Experimental Design, Materials and Methods

**Fig. 4:**
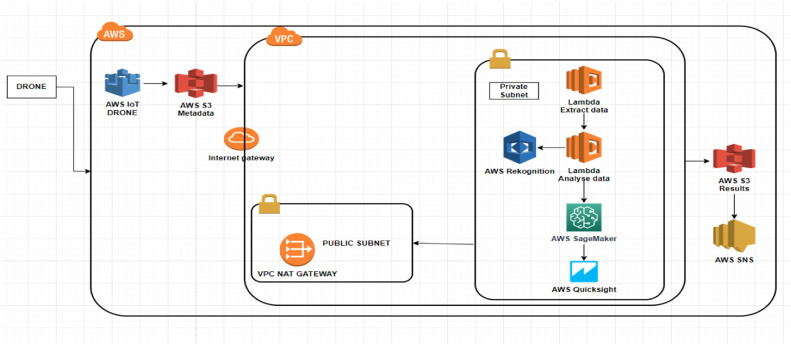
Steps involved in implementing PV module fault detection and analyzing algorithms.

There are several interconnected steps involved in implementing the automated PV module fault detection and analysis algorithms before deployment. Fig. 4 shows all the several interconnected steps involved in implementing the PV module fault detection and analyzing algorithms.

### 4.1 Data acquisition

**Fig. 5:**
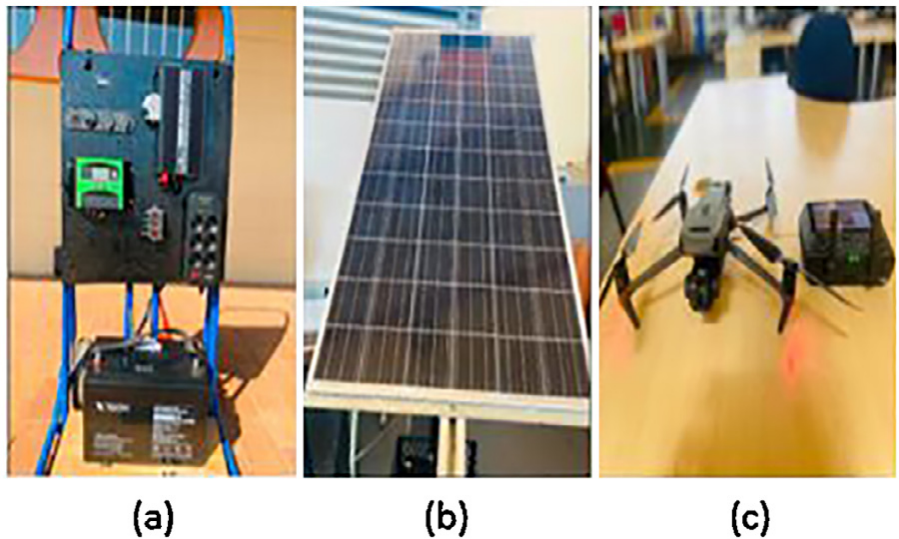
DISPLAY laboratory at Tshwane University of Technology showing implemented (a) PV module (b) Solar panels (c) DJI Mavic 3 Thermal equipped with an integrated thermal camera.

DJI Mavic 3 Thermal's high-resolution thermal and visual imaging capabilities were utilized to capture the PVMD dataset on September 5, 2024, and this was by taking faulty PV module images from the Soshanguve South Campus, Tshwane University of Technology, South Africa. The dataset has a total of 1000 images of three permanent and temporal anomalies, namely hotspots, cracks, and shading. DJI Mavic 3 Thermal coupled with its advanced flight features such as compact and portable, 4/3 CMOS wide camera, 56× hybrid zoom, 640 × 512 px thermal camera, 45-min max flight time, DJI O3 Enterprise transmission, centimeter-level positioning with RTK, and high-volume loudspeaker [4], makes it an excellent tool for precise and efficient inspections of PV systems. Fig. 5 depicts the Digital Image and Signal Processing (DISPLAY) laboratory at Tshwane University of Technology showing implemented (a) PV module (b) Solar panels (c) DJI Mavic 3 Thermal equipped with an integrated thermal camera, demonstrating its capability for advanced aerial thermal imaging and inspection tasks. The DJI Mavic 3 Thermal, equipped with its high-resolution thermal camera, was employed for capturing thermal images. In the DISPLAY laboratory, we simulated external defects on the system by placing materials such as polystyrene behind the PV panel, adhesive paper on the front glass, and a small piece of gum. We also utilized an input image from an online source to further test the system. The laboratory experiment performed on the dataset lasted one week. Because the data comes from a single-day collection and one week laboratory experiment, it makes the data more suitable for testing algorithms designed for fault detection.

### 4.2 Pre-processing and analysis

The dataset includes both permanent and temporal anomalies such as hotspots, cracks, and shading. The RGB images which were originally of different dimensions due to unfriendly and unstable environmental conditions were trimmed to size 512×512×3 (3 keeps the color information) for uniformity of the images using Microsoft Snipping Tool (MST] for further processing. Two buckets were created: one for uploading images and another for storing the results after the images were processed using a Machine Learning (ML) component, Rekognition. A Lambda function handles the processing by invoking Rekognition, which is a ML tool. The processing of thermal and visual data was carried out using Python 3.x on a Windows 11 Home environment equipped with an Intel Core i5-4210M CPU @ 2.60 GHz and 8 GB of RAM. Python, along with libraries such as Flask, OpenCV, NumPy, and Matplotlib, was used to analyze and process the images. The libraries were installed using pip commands, facilitating the implementation of image processing tasks such as thermal anomaly detection.

### 4.3 Data augmentation

Augmentation of data in ML task is an effective technique of increasing the quantity of dataset and creating diversity of data representations for the training and testing of a model and for a balanced classification. Moreover, the techniques of data augmentation reduce overfitting and increase performance of the designed model [5]. However, acquisition of such magnitude of data is a factor of some conditions, namely condition of the atmosphere, condition of the sunlight, condition of the environment, absence of thermal anomaly during the data acquisition, etc. The above-mentioned issues are best resolved by data augmentation techniques. In this article, knowing that the proposed method for automated PV module fault detection and analysis in large PV systems depends heavily on availability of large quantity of data; we applied basic data augmentation techniques for the enhancement of the dataset as specification demands. This work employed basic data augmentation techniques, namely geometric transformation, color-based transformations, illumination transformation, noise injection, etc. [5].

### 4.4 Feature extraction and graph generation for visual representation

ML based tools may be employed to carry out feature extraction and graph generation effective detection and analysis of faulty solar cells in PV module. Extraction of features from an image by ML model is an important phase in any object detection and segmentation task including the efforts put to classify the individual object according to their instance or semantic class [6]. The primary aim of feature extraction is to extract important information about the object in the image for further processing; and this is automatically performed without any assistance from human expert, thanks to techniques of advanced ML implemented in the learning algorithm. As a ML service for building, training and deploying ML models at scale using any of the tools such as notebooks, debuggers, profilers, pipelines, and many more-all in one, we employed SageMaker and other ML services to process the dataset presented including the augmentation techniques aforementioned, and to generate a graph after retrieving results from the result bucket, creating a visual representation of the metrics.

The services used in this work and their functions are presented as follows.

(1) AWS IoT Core:
  - Function: Enables the secure transmission of images captured by drones to the cloud.
  - Role: Acts as a communication bridge between drones and the cloud, ensuring images are securely and reliably transmitted.
(2) Amazon S3 (Simple Storage Service):
  - Function: Offers scalable storage for raw images and processed data.
  - Role: Serves as the main storage location for all project-related data, including images captured by drones and processed image results.
(3) AWS Lambda:
  - Function: Executes image processing tasks, such as detecting defects and performing morphological operations.
  - Role: Processes images in response to triggers (e.g., new image uploads), running code without the need to manage servers.
(4) Amazon Rekognition:
  - Function: Detects visual defects, such as hotspots, cracks, and shadings issues in solar panels.
  - Role: Provides AI-powered image analysis, automating the detection of issues in solar panels.
(5) OpenCV on Lambda:
  - Function: Performs morphological operations like erosion, dilation, opening, and closing on images.
  - Role: Enhances image processing capabilities by applying filters and transformations that help better visualize and analyze defects.
(6) Amazon SageMaker:
  - Function: Provides advanced analytics and generates metrics and graphs based on processed images.
  - Role: Runs ML models or other advanced analytical operations to derive insights from image data.
(7) Amazon QuickSight:
  - Function: Visualizes data through dashboards and reports.
  - Role: Creates interactive visualizations and dashboards that allow users to see analysis results and metrics in a clear, actionable format.
(8) AWS SNS (Simple Notification Service):
  - Function: Sends notifications upon the completion of image processing tasks.
  - Role: Keeps users informed about the processing status, providing timely updates and alerts.
(9) AWS DynamoDB Database: Stores processed data and analysis results.

## Limitations

The data that made up the PVMD dataset described in this article are RGB images which were originally of different dimensions due to drone employed, unfriendly and unstable environmental conditions under which they were collected. This negatively affected the quality and size of the dataset.

## Ethics Statement

The authors confirm that the work in this article does not involve human subjects, animal experiments, or any data collected from social media platforms.

## CRediT Author Statement

Rotimi-Williams Bello, Pius A. Owolawi, Etienne A. van Wyk & Chunling Du: Investigation of data collection methods, Methodology, Conceptualization; Rotimi-Williams Bello: Writing - Original draft preparation; Rotimi-Williams Bello: Writing - review & editing; Pius A. Owolawi, Etienne A. van Wyk & Chunling Du: Data Verification.

## Data Availability

Mendeley DataPhotovoltaic module dataset for automated fault detection and analysis in large photovoltaic systems using photovoltaic module fault detection (Original data).
